# Non-adherence to anti-retroviral medication in Shiraz, 2014: a cross sectional study

**DOI:** 10.4314/ahs.v18i2.24

**Published:** 2018-06

**Authors:** Nasrin Motazedian, Mehrab Sayadi, Ali Firoozbakhtian

**Affiliations:** 1 Shiraz Transplant Research Center, Shiraz University of Medical Sciences, Shiraz, Iran; 2 Student Research Committee, Medical School, Shiraz University of Medical Sciences, Shiraz, Iran; 3 Shiraz HIV/AIDS Research Center, Shiraz University of Medical Sciences, Shiraz, Iran

**Keywords:** Prevalence, adherence, anti-retroviral medication, HIV

## Abstract

**Background:**

Medication adherence is a dynamic and complex behavioral process, which is strongly influenced by personal, social and environmental factors.

**Objectives:**

To determine the prevalence and factors affecting non-adherence to medication among HIV-infected patients.

**Methods and materials:**

**Design:**

A cross-sectional study.

**Setting:**

Voluntary Counseling and Testing Center (VCT), Shiraz, Fars province, in the South of Iran.

**Patients:**

Among HIV-positive patients who received anti-retroviral therapy, 214 adult patients were selected through convenience sampling. Their medication adherence was checked by interview and counting the pills on visits during two months. Clinical and laboratory data were obtained from the patients' records.

**Results:**

Non-adherence and adherence groups included 30.4% (65) and 69.6% (149) of the patients, respectively. The mean age of patients was 40.80±7.77 years, and ranged from 20 to 65 years. Majority of cases (65%) were male. A significant relationship was found between non-adherence to medications and the variables of transmission method, marital status, housing status, and CD4, but there was no significant relationship with gender.

**Conclusion:**

The prevalence of medication adherence was similar to other regions with limited financial resources. To increase patient's medication adherence, they should be exposed to motivational interventions to promote their drug consumption, social and occupational support.

## Introduction

**Background:** Anti-retroviral medication suppresses viral replication in HIV-infected individuals. Evidence shows that broader and earlier commencement of ART can reduce HIV rate^1^. High level drug adherence for a long period of time is necessary to suppress viruses^2^. Medication adherence is a dynamic and complex behavioral process, strongly influenced by the personal, social and environmental circumstances^3^.

Medication non-compliance was reported as 45% in the USA^4^. Other studies in Africa and Asia have shown that more than 70% of HIV-positive young adults and adolescents receiving anti-retroviral medication were adherent to their prescription, but adherence rate was lower in Europe and North America (50–60%). These differences, which are multifactorial, represent the difference between centralized and distributed epidemic as well as access to health systems and resources^5^.

There are significant obstacles to medication adherence amongst HIV-infected individuals and occurrences such as food shortages, financial constraints, forgetfulness, fear of being labeled, and stigma as well as the psychosocial aspects of the illness (e.g.anxiety and depression) directly or indirectly affect their behavior.

Higher trust or satisfaction rate with the HIV caregiver, lower daily dose frequency and not having depression symptoms were strongly linked to higher adherence in low and medium Human Development Index countries than in high Human Development Index countries^6^,^7^,^8^.

Among the established indices, high social status (income, education, employment status) was associated with higher adherence to anti-retroviral drugs^9^. Adherence may also vary according to gender^10^.

Among the psychosocial factors, certain aspects of the relationship between patient and care providers, such as the service provider's ability and skills, trust, open communication, willingness to consume their medication, and general satisfaction were significantly related to higher rates of adherence^11^.

Another study showed non-adherence key factors: being away from home, being busy and forgetfulness^12^. However, systematic review of the available evidence does not provide conclusive evidence of a clear relationship between socio-economic status and adherence to anti-HIV drugs in young adults infected with HIV/AIDS in low and middle income countries. It seems that a positive relationship exists between the components of socio-economic status (income, education, and employment) and adherence to anti-retroviral medication^13^.

However, compelling a patient to take medicine for the rest of his/her life without forgetting a dose is challenging. Medication adherence factors might vary from one patient to another and from time to time.

**Objectives:** The present study aimed to determine the prevalence and factors affecting medication adherence among HIV-infected patients referred to Voluntary Counseling and Testing (VCT) center of Shiraz, Iran.

## Methods and materials

**Study design:** This was an analytical cross-sectional study.

**Study setting:** Our study was conducted in Shiraz VCT center. Shiraz VCT center is the only center in Fars province (in the South of Iran) that provides pharmaceutical and healthcare consultation services to these patients. Hence, all identified HIV/AIDS individuals in Fars province attend this center. We collected data during two months of March–April 2014. Patients' medication adherence was checked through interview and counting of the pills during their visits in each month. This study was approved by the local Ethics Committee of the Shiraz University of Medical Sciences.

**Study size:** The minimum sample size was calculated according to our objectives, and the non-adherence prevalence of 30% from a previous study with 95% confidence interval, error estimation of 5%. The formula

$$n=\frac{{\left(z_{1-\frac{n}{2}}\right)}^{1}p(1-p)}{{\left(d\right)}^{2}}$$

was used regarding finite population correction, $m=\left[\frac{n}{1+\frac{n}{N}}\right]$ [m=224 person, (N=730)].

**Figure 1:**
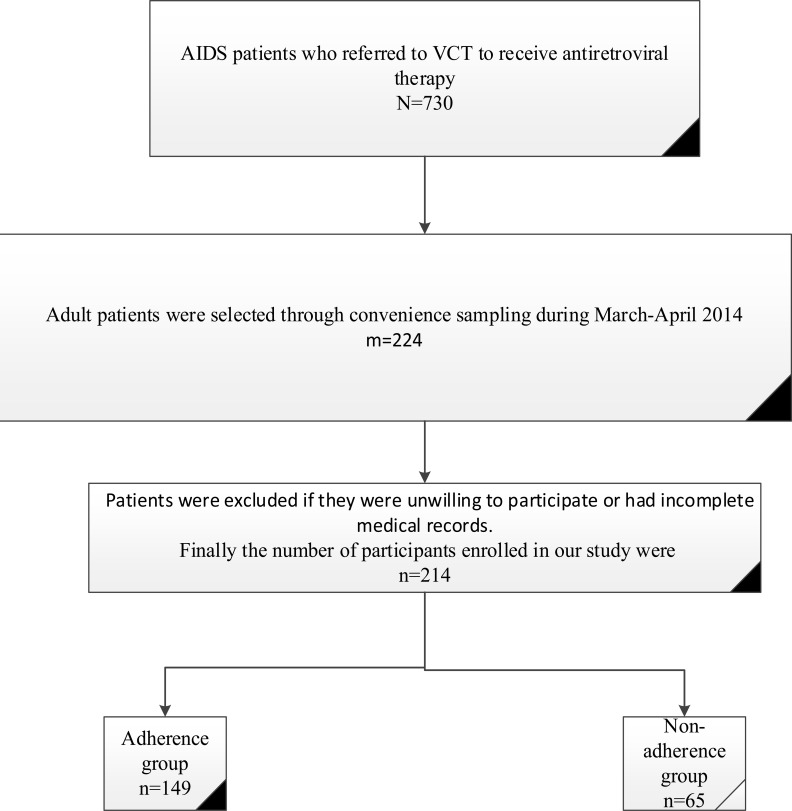
Flow chart for selection of study participants

**Participants:** Among HIV-infected patients referred to Voluntary Counseling and Testing (VCT), center of Shiraz, Iran, 224 adult patients were selected through convenience sampling. Finally, 214 patients with complete medical records and good cooperation were enrolled in this study. Convenience sampling was used, since most of those who participated were drug addicts and they were not willing to participate in our study. In order to have comprehensive coverage of all the patients, samples were collected every day during opening hours of the center (Figure 1).

The study objectives were explained and participants were assured that their information will remain confidential. Those who had the inclusion criteria and were willing to participate in the study signed a written informed consent and were enrolled in the study. All adolescents were excluded, and the next person who met the inclusion criteria was recruited.

**Variables:** The form for data collection included sociodemographic questions such as age, gender, and marital status, type of accommodation, place of residence, level of education, income level, and employment. Factors related to compliance included: assistant in drug consumption, access to daily meals, methadone consumption, and travelling time to the center. Mode of HIV transmission, hospitalization, CD4, BMI, and clinical staging, were obtained from the patient's medical records.

**Bias:** Our study was a cross sectional study and was susceptible to the effects of selection bias and recall bias. The potential for selection bias was minimized by extending the study time daily during the opening hours of the center. To minimize recall bias, we excluded patients who could not correctly remember their medical consumption and/or their medical records were incomplete.

**Statistical methods:** Quantitative data was reported using Mean ± SD and qualitative data was expressed as percentages. Missing data was ignored and complete data was analyzed using chi-square and t- test. Also, we used logistic regression model to control the confounders using SPSS-18. The significance level was considered at 5% for all tests.

## Results

**Participants:** This study was designed to investigate factors influencing non-compliance to medication in patients with HIV/AIDS in Shiraz, Iran. A total of 214 patients were divided into two groups according to medication adherence or non-adherence, and were compared.

Based on the results, 30.4% (65) of patients were non-adherent and 69.6% (149) showed good adherence to their medication protocols. The most important reason for discontinuation of medication was the patients' unknown personal reasons (n=60, 61.2%), from the non-adherence group. In 168 participants (78.5%), Zidovudine (AZT), Lamivudine (3TC), and Efavirenz (EFV) were the first drug of choice.

**Descriptive data:** The patient's age range was 20–65 years with an average of 40.80±7.77 years. The majority of cases (65%) were male. Variables of age, gender, employment, housing, marital status, and education level in both groups are summarized in Table 1. Most of the subjects (53.3%) were married, and 32.7% had a middle school degree.

**Table 1:** Evaluation and comparison of demographic and socio-economic characteristic in HIV-Infected adults receiving anti-retroviral therapy in Fars province, 2014

Variables		Group		Statistical index	*p*-value
		Non-adherence	Adherence		
Age (Mean ± SD)		41.68±6.6	40.42±8.22	1.09	0.276
Gender (%)	Female (n=75)	8 (12.3)	67 (45)	21.2	<0.001*
	Male (n=139)	57 (87.7)	82 (55)		
Marital Status (%)	Single/Divorced/Widowed (n=99)	43 (67.2)	56 (37.6)	15.7	<0.001*
	Married (n=114)	21 (32.8)	93 (62.4)		
Education (%)	Illiterate (n=9)	1 (1.5)	8 (5.4)	4.5	0.21
	Primary school (n=69)	26 (40)	43 (28.9)		
	Middle school (n=70)	22 (33.8)	48 (32.2)		
	High school and higher (n=66)	16 (24.6)	50 (33.6)		
Location (%)	City (n=190)	61 (32.1)	129 (67.9)	2.4	0.121
	Village (n=24)	4 (16.7)	20 (83.3)		
Housing (%)	Personal house (property) (n=39)	5 (7.7)	34 (22.8)	17.8F	<0.001*
	Personal house (leased) (n=93)	22 (33.8)	71 (47.7)		
	Living with a family (n=79)	37 (56.9)	42 (28.2)		
	Other (n=3)	1 (1.5)	2 (1.3)		
Occupation (%)	Steady (44)	10 (15.4)	33 (22.8)	6.16	0.046*
	Non-steady (n=34)	6 (9.2)	28 (19.3)		
	Unemployed (n=133)	49 (75.4)	84 (57.9)		
Income status (%)	Less than 3000000 Iranian Rials (100 USD†) (n=23)	4 (17.4)	19 (82.6)	0.393	0.942
	3000000 to 5000000 Iranian Rials(100–166 USD) (n=24)	4 (16.7)	20 (83.3)		
	5000000 to 10 million Iranian Rials (166–332 USD (n=27)	6 (22.2)	21 (77.8)		
	10 to 15 million Iranian Rials (332–497 USD) (n=4)	1 (25)	3 (75)		

* Statistically significant

† United States Dollar (USD)

**Outcome data:** According to Table 1, the variables of gender, marital status, housing, and occupation had a significant association with non-adherence to medication. This was more common amongst men and in unmarried (single, divorced, or widowed) than married couples. Those with unsteady occupations adhered better to their medication than the unemployed participants.

Time of access to VCT center for people in the non-adherence and adherence groups was 54.38 ± 58.9 and 69.26 ± 78.26 minutes, respectively (p=0.110 and t=1.608), but the difference was not statistically significant.

History of hepatitis C and hospitalization was reported in 30.5% and 55.2% (116 patients), respectively. Non-adherence patients with history of hospitalization were more than those without hospitalization history (35.3% vs. 22.3%), and this difference was statistically significant (p=0.040, X2=4.22). (Table 2)

**Table 2:** Comparing factors associated with adherence to anti-retroviral therapy, in Fars province, 2014

Variables		Group		Statistical index	*p*-value
		Non-adherence	Adherence		
Travelling time to the center		54.38 ± 58.9	70.5 (83.26)	T=1.608	0.110*
Mode of HIV transmission	Drug injection (n=43)	24(38.1)	19 (12.9)	X^2^ = 23.8	<0.001*
	Sexual contact (n=18)	3 (4.8)	15 (10.2)		
	Drug injection and sexual contact (n=82)	26 (41.3)	56 (38.1)		
	Spouse of the patient (n=52)	6 (9.5)	46 (31.3)		
	Other (n=15)	4 (6.3)	11 (7.5)		
Hospitalization	Yes (n=116)	41 (66.1)	75 (50.7)	X^2^=4.22	0.040*
	No (n=194)	21 (33.9)	73 (49.3)		
Assistance in drug consumption	Family (n=66)	26 (39.4)	40 (60.6)	X^2^=5.2	0.074
	Patient (n=132)	35 (26.5)	97 (73.5)		
	Family and patient (n=14)	2 (14.3)	12 (85.7)		
Access to regular food	Yes (n=168)	53 (81.5)	115 (78.2)	X^2^ = 0.3	0.584
	No (n=44)	12 (18.5)	32 (21.8)		
Methadone consumption	Yes (n=69)	40 (61.5)	29 (21)	X^2^=32.34	<0.001*
	No (n=134)	25 (38.5)	109 (79)		
CD4		163.74±226.77	202.1±326.55	t=3.5	<0.001*
BMI		22.26±4.9	23.32±4.1	T=1.57	0.116
Clinical staging	1(n=86)	21 (32.3)	65 (43.9)	X^2^=5.49	0.139
	2(n=85)	54 (36.5)	31 (47.7)		
	3(n=37)	24 (16.2)	13 (20)		
	4(n=5)	0 (0)	5 (3.4)		
Social support score		29.3±4.6	28.40±3.58	X^2^=1.51	0.132

* Statistically significant

Length of hospital stay was different among participants (ranging from 1 to 180 days with an average of 20.95 ± 37.36 days). They were admitted for various reasons, with surgery being the most common (24.5%).

The majority of patients in both groups (n=132, 62.3%) stated that no one assisted them in drug consumption, which had no significant association with non-adherence to medication. In addition, 202 patients (94%) stated that medical staff had explained drug consumption methods and their possible side effects.

Non-adherence was lower in those who took methadone (58%) than those who did not use it (18.7%), which had a significant association with non-adherence to medication (p=<0.001, X2=32.34) (Table 2).

CD4 ranged from 11 to 688 with a mean of 179.9 ± 115.8, with a significant difference in the two groups. BMI of the participants varied from 12.4 to 37.7 with a mean of 23.2 ± 4.38, but was not significant.

In the studied patients, 40.4%, 39.9%, 17.4%, and 2.3% were in clinical stages of 1, 2, 3, and 4, respectively, which had no significant difference in terms of adherence and non-adherence to medication. (Table 2)

The social support score ranged from 23 to 51 with a mean of 28.67 ± 3.94, but that was not significant.

**Main results:** To evaluate adherence to medication, the multiple logistic regression analysis was used and a significant relationship was found between the non-adherence group and the variables of transmission method, marital status, housing, and CD4. However, the data in this study did not show any significant relationship with gender. Among the significant variables, transmission method and marital status had a reverse relationship with non-adherence (Table 3).

**Table 3:** The socio-demographic, behavioral, and clinical predictors of non-adherence to antiretroviral therapy, in Fars province, 2014

Variables	Adjusted OR	*p*-value	(95% CI)
**Mode of HIV transmission**			
Drug injection	Ref		
Sexual contact	0.068	0.005	0.010–0.445
Drug injection and sexual contact	0.297	0.011	0.117–0.755
Spouse of the patient	0.505	0.509	0. 066–3.836
Other	0.637	0.594	0.122–3.335
**Marital status**			
Single/divorced/widowed	Ref		
Married	0.181	<0.001	0.073–0.444
**Housing**			
Personal (property)	Ref		
Personal (leased)	3.723	0.046	1.025–13.530
Living with family	7.080	0.002	2.025–24.748
Other	1.251	0.872	0.082–19.004
**Gender**			
Female	Ref		
Male	4.800	0.074	0.860–26.804
**CD4**	0.996	0.001	0.994–0.998

## Discussion

**Key results:** In this study, the prevalence of adherence was evaluated and the factors involved in both groups were compared. The rate of non-adherence in the present study was 30.4%.

Multiple logistic regression analysis showed a significant relationship between non-adherence to medication and the variables of transmission method, marital status, housing, and CD4, however, the data did not show a significant association with gender.

**Interpretation:** According to literature, medication adherence is higher in countries with limited financial resources than the advanced countries^5^,^14^,^15^,^16^. Studies from countries with strong financial resources have shown that less than 50% of patients take all anti-retroviral drugs on time as directed by caregivers^14^. Nonetheless, these results were lower than the results of our study. The majority of the participants in our study stated that medical staff had explained how to consume drugs, the advantages, and the side effects of the medications before initiation of the therapy, which could be the reason for their adherence to medication. In addition, VCT center in Shiraz supplies free HAART (highly active anti-retroviral therapy), physician visit, and CD4 count test for all HIV/AIDS patients. Adherence rate is related to resources and availability of healthcare facilities. For example, HIV screening and providing ART (anti-retroviral therapy) are widespread, with door to door testing and care in underprivileged regions. It is also free of charge for disadvantaged people^5^. This could also be a good reason for the higher adherence rate in these regions.

In most of the women who were infected through their husbands, the gender variable had a significant relationship with medication adherence using Chi-square analysis. On the contrary, according to logistic regression analysis no significant relationship was found. Another study in Shiraz showed that even though women referred later to health care centers (mostly in the AIDS phase), but they visited more regularly and complied better with medication in comparison to men. However, survival rates did not differ between men and women^17^. Another study showed that men had less adherence in taking ART medication^18^. A study in India did not find a significant relationship between gender and drug adherence, which could be due to small sample size^15^. It could also be due to the different levels of stigma and violence against women in different regions.

Consumption of methadone was reported more in the non-adherence group, and drug abuse could be the reason. Among the non-adherence group in the present study, 38.1% of patients were intravenous drug users.

In logistic regression analysis it was shown that adherence to medication was higher in those infected through sexual contact or drug use in comparison to drug users group. It seems that these people were non-adherent to medication due to drug addiction. A study showed non-adherence is more in people who use drugs^19^. Another study that compared adherence to medication among intravenous drug users (IDUs) and non-intravenous drug users infected with HIV/AIDS showed that injecting drug users had a higher rate of non-adherence^20^.

It is unclear as to why those with high CD4 count had higher adherence to medication. A study in the USA obtained a higher mean CD4 in patients with medication adherence than those without medication adherence, and this difference was significant. This was consistent with the results of the present study^21^. But the results of a study in South India showed that people with CD4 of more than 500 cells per microliter and long-term drug users had less adherence to medication; this might be due to boredom of taking a drug for a long period of time^22^. One explanation is that people with higher CD4 felt better and those who were health illiterate did not take their medication correctly.

Married individuals have a better chance of medication adherence than single people.Living alone and being unemployed were also factors involved in non-adherence to medication in our study. A study in Vietnam obtained similar results^23^. Another study showed that living alone led to lower adherence^24^. Being a couple can help people to remind each other to consume their medications and accompany each other to centers; hence, better adherence.

A systematic review reported a significant relationship between the elements of SES (education, income, and occupation) and adherence to medication^9^. The majority of the participants in our study did not answer the income question rendering it un-analyzable, but occupation had a significant relationship between groups, and the majority of patients in the non-adherence group were unemployed. The housing situation also had a significant relationship with their adherence. Most people with adherence to medication had private homes, but most people in the non-adherence group were either living with family or had to pay rent. It seems that stable conditions including married life and having a home might be helpful in their adherence.

In the present study, no significant relationship existed between social support and medication adherence. In general, factors determining socio-economic status are related to cultural, economic, and geographic factors. For this reason, studies achieved different outcomes due to different contexts. Adherence to medication varies in different countries. It is suggested that geographical location should be considered.

The present study was the first research performed on people with HIV/AIDS in Shiraz, in which the prevalence of medication adherence and its affecting factors were evaluated. This study can be a benchmark for other interventional and longitudinal studies.

## Limitations

This is a cross sectional study, which was conducted in one center and might not be applicable for other regions.

## Conclusion

The rate of medication adherence in this study was similar to other regions with limited financial resources. Therefore, to increase adherence, patients should be exposed to motivational interventions for drug consumption and social and occupational support. Medication and medical care should be widespread, provide more over the phone and social workers services. Since medication adherence is a dynamic process and changes over time, it is suggested to design a longitudinal study to achieve better results and higher adherence to drug therapy through monitoring medication and understanding the factors involved in non-adherence in bigger sample sizes over time.
